# Selectin Binding Sites Are Involved in Cell Adhesive Properties of Head and Neck Squamous Cell Carcinoma

**DOI:** 10.3390/cancers11111672

**Published:** 2019-10-28

**Authors:** Ursula Valentiner, Jillian Knips, Ralph Pries, Till Clauditz, Adrian Münscher, Guido Sauter, Barbara Wollenberg, Udo Schumacher

**Affiliations:** 1 Institute of Anatomy and Experimental Morphology, University Cancer Center Hamburg, University Medical Center Hamburg-Eppendorf, Martinistrasse 52, 20246 Hamburg, Germany; u.schumacher@uke.de; 2 Department of Oral and Maxillofacial Surgery, University Medical Center Hamburg-Eppendorf, Martinistrasse 52, 20246 Hamburg, Germany; jknips@uke.de; 3 Department of Ear, Nose and Throat, University Hospital Schleswig-Holstein, Ratzeburger Allee 160, 23538 Lübeck, Germany; Ralph.Pries@uksh.de (R.P.); Barbara.Wollenberg@uksh.de (B.W.); 4 Institute of Pathology, University Medical Center Hamburg-Eppendorf, Martinistrasse 52, 20246 Hamburg, Germany; t.clauditz@uke.de (T.C.); g.sauter@uke.de (G.S.); 5 Department of Otolaryngology, University Medical Center Hamburg-Eppendorf, Martinistrasse 52, 20246 Hamburg, Germany; a.muenscher@uke.de

**Keywords:** head and neck squamous cell carcinoma, xenograft model, SCID mouse, metastasis, cell adhesion molecules, selectin

## Abstract

The formation of distant metastases often determines the fate of patients with head and neck squamous cell carcinoma (HNSCC). The expression of cell adhesion molecules (CAMs) and their ligands of the leukocyte adhesion cascade has been associated with metastatic competence in several malignant entities. In this study, human HNSCC cell lines were analyzed in vitro and in a spontaneous metastatic xenograft model. Immunohistochemical analyses of several CAMs were performed on xenograft tumors and tissue microarrays (TMA) from 453 patients with head and neck squamous cell carcinomas with full histo-pathological and clinical follow-up data. UTSCC 24A and 24B cells bind to E-selectin in vitro, show E-selectin dependent binding to human umbilical vein endothelial cells (HUVECs), and express sLeX. All HNSCC cells engrafted into severe combined immunodeficient (SCID) mice, and UTSCC 24A cells formed sporadically spontaneous lung metastases. The expression of CAMs varied between the cell lines, but a correlation between tumor growth and metastatic potential did not exist. None of the CAMS or their ligands could be identified to be of prognostic relevance in the TMA study. The in vitro results indicate that E-selectin and sLeX are involved in the adhesion of HNSCC cells to endothelium. However, specific prognostic markers chosen from the leukocyte adhesion cascade for HNSCC were not identified.

## 1. Introduction

Improvement in the clinical management of head and neck squamous cell carcinoma (HNSCC) patients has resulted in loco-regional control of the primary tumor; however, long-term survival has not significantly changed due to the formation of distant metastases [1]. Metastasis formation is a complex process which cannot be completely modeled in vitro. Therefore, clinically relevant animal models of spontaneous metastases are absolutely essential for HNSCC metastasis research. Within the metastatic process, adhesive properties of the tumor cells play a significant role in the formation of distant metastases. Therefore, the downregulation of homotypic cell-cell adhesion is required for the detachment of metastatic cells from the primary tumor, while the upregulation of cell adhesion molecules (CAM) mediating the attachment of cancer cells to endothelial cells is a precondition for extravasation at the future metastatic site. Regarding this step, it is assumed that tumor cells mimic mechanisms used by leukocytes and platelets in inflammatory processes. This so called leukocyte adhesion cascade consists of several sequential steps and provided the rationale of several reports investigating the adhesion properties of cancer cells other than HNSCC [2,3,4,5,6,7]. The family of selectins initiates the adhesive process of leukocytes by trapping cells from the bloodstream and slowing them down [2]. The initially loose adhesion causes integrin activation and full arrest of the cells to activated endothelial cells and is followed by the last step of the cascade, the transendothelial migration (TEM) [3,8,9,10]. Though it is controversial whether selectin mediated rolling is necessary for the TEM of cancer cells and molecular mechanisms of leukocyte and tumor cell extravasation can differ, CAMs mediating the contact with endothelial cells are potentially the same [11,12]. Therefore, recent studies have reported that selectins and integrins play a role in the formation of metastases of various cancer types [11,13,14].

In this report, we compare the ability of human HNSCC cells to form primary tumors and spontaneous metastases in the lungs of severe combined immunodeficient (SCID) mice. The expression of CAMs is analyzed in vitro, in mouse xenografts, and in tumor tissue microarrays (TMA) including patient material of 453 cases of head and neck squamous cell carcinomas in order to identify potential prognostic markers which allow for a meaningful monitoring of the HNSCC patient.

## 2. Results

### 2.1. Flow Cytometry of in Vitro Grown Cells

**Figure 1:**
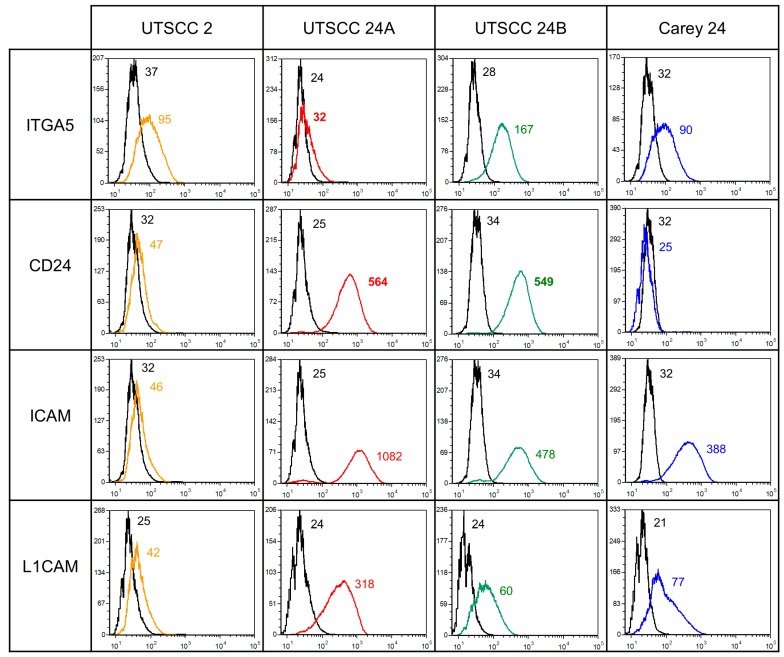
Representative flow cytometric histograms of selected cell surface glycoproteins, whose expression differed between the tested cell lines. Colored lines present specific mAb binding and black lines indicate the corresponding isotype control. Numbers show the mean fluorescence intensity (MFI). ICAM and L1CAM were highly expressed by UTSCC 24A and 24B and Carey 24, but lowly expressed by UTSCC 2. ITGA5 expression was the highest in UTSCC 24B, followed by UTSCC 2 and Carey 24, and was the lowest in UTSCC 24A. CD24 was detected on UTSCC 24A and 24B, but hardly on UTSCC2 and Carey 24 cells.

ITGA6, ITGB1, CD46, EpCAM, ALCAM, ITGAV, ITGB4, E-cadherin, and CD44 were detected on all analyzed HNSCC cells, without considerable differences between the cell lines. ITGA5 was observed on UTSCC 24B (139 mean fluorescence intensity—MFI), UTSCC 2 (58 MFI), and Carey 24 (58 MFI) cells, but hardly on UTSCC 24A (8 MFI) cells (Figure 1). ICAM expression was highest on UTSCC 24A (1057 MFI), followed by UTSCC 24B (444 MFI) and Carey 24 (366 MFI) cells, whilst UTSCC 2 (14 MFI) cells slightly expressed ICAM (Figure 1). CD24 was expressed by UTSCC 24A (538 MFI) and UTSCC 24B (515 MFI), and levels of CD24 were negligible on UTSCC 2 (15 MFI) and Carey 24 (0 MFI) cells (Figure 1). The MFI of L1CAM expression was 294 in UTSCC 24A, 56 in Carey, 44 in UTSCC 24B, and 17 in UTSCC 2 cells (Figure 1). CD162, CD11a, CD11b, ITGB3, ITGB5, and ITGB7 were not or only slightly detected on HNSCC cells.

### 2.2. Laminar Flow Assay, Selectin Binding, and Canonical Selectin Ligand Expression in Vitro

**Figure 2:**
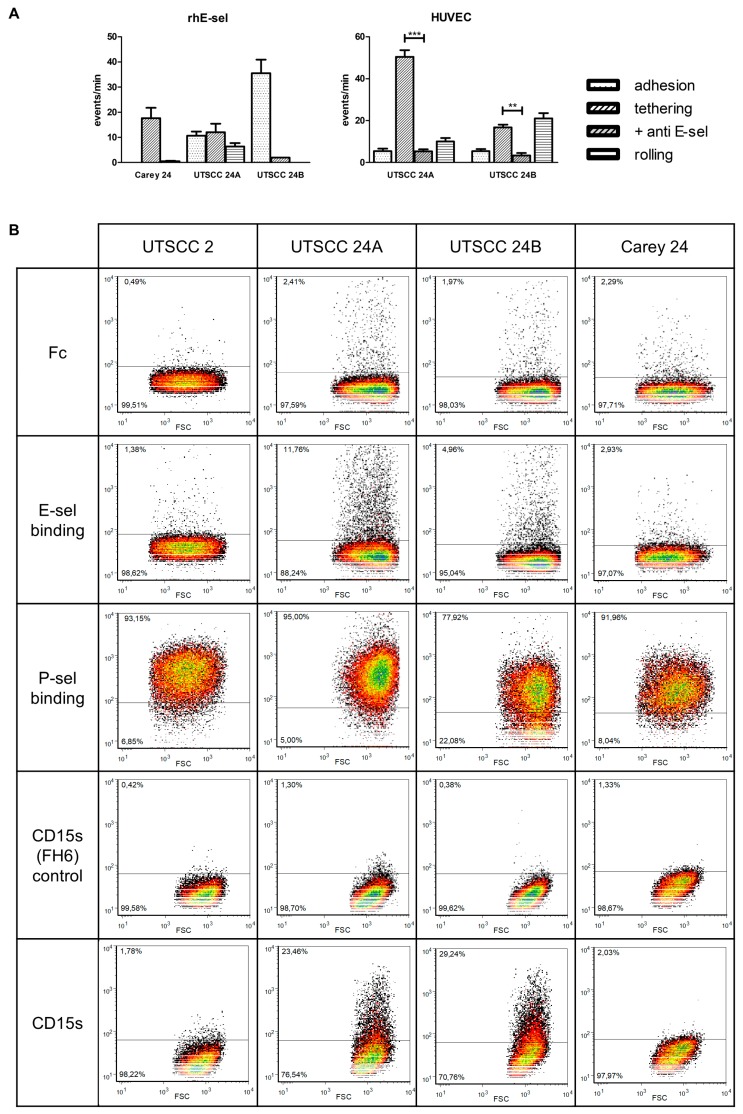
(**A**) Cell flow analysis of human head and neck squamous cell carcinoma cell lines (HNSCC) cells on rhE-selectin-Fc-chimera and on confluent monolayers of IL-1α-stimulated and unstimulated human umbilical vascular endothelial cells. UTSCC 24B cells most strongly adhered to rhE-sel, and UTSCC 24A cells showed the highest number of adhesive events for stimulated HUVECs. Incubation with the adhesion blocking anti-E-selectin mAb significantly reduced the tethering of UTSCC 24A and tethering and rolling of UTSCC 24B cells to HUVECs (* *p* < 0.05, ** *p <* 0.01, *** *p* < 0.001). (**B**) Representative flow cytometric histograms of selectin binding and canonical selectin ligand expression. All HNSCC cells bound to rhP-selectin, but only UTSCC 24A and B cells bound to rhE-selectin. HNSCC cells did not express CA19-9 (sLeA), but 23% of UTSCC 24A and 29% of UTSCC 24B cells expressed CD15s (sLeX).

**Figure 3:**
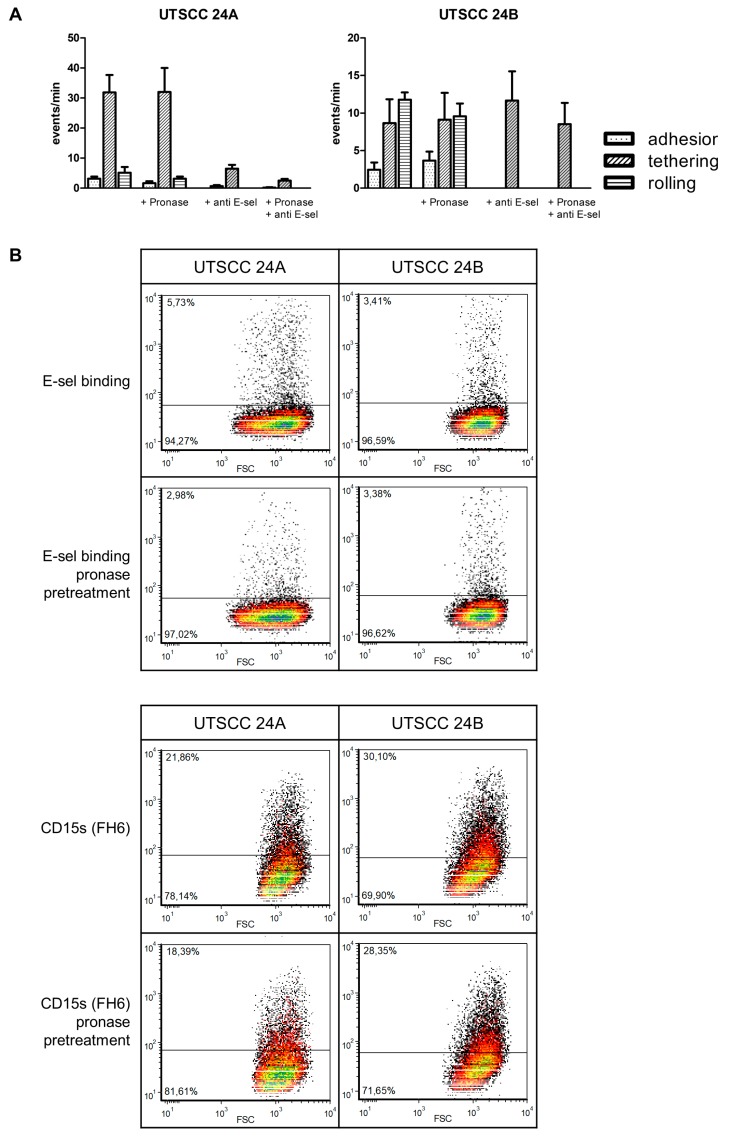
(**A**) Binding of UTSCC 24A and 24B cells to HUVECs with and without pre-incubation with the E-sel antibody was not significantly influenced by proteolytic pretreatment of tumor cells with pronase. (**B**) Pronase treatment slightly reduced static E-selectin binding and CD15s (sLeX) expression in UTSCC 24A, but not in UTSCC 24B cells.

UTSCC 24B cells most strongly adhered to the rhE-sel fusion protein under shear flow conditions, presenting 36 firm adhesive events followed by UTSCC 24A cells (29 events) which showed firm adhesion, tethering, and equally distributed rolling. Carey 24 cells adhered to a lesser extent (18 events) and were predominantly tethered on the rhE-sel fusion protein (Figure 2A). UTSCC 2 cells rarely adhered to the rhE-sel fusion protein (1 event). Cells of all HNSCC cell lines did not adhere to the rhP-sel fusion protein under flow. UTSCC 24A and UTSCC 24B cells adhered considerably to stimulated HUVECs (69 and 43 events, respectively, Figure 2B). Tethering was the predominant event of UTSCC 24A and rolling was the predominant event of UTSCC 24B cells. Adhesive events were significantly inhibited by pre-incubation with the anti E-sel antibody in both cell lines (tethering: UTSCC 24A 54 vs. 5, *p* < 0.001 and UTSCC 24B 17 vs. 3, *p* < 0.05; rolling: UTSCC 24B 21 vs. 0, *p* < 0001, Figure 2A). In contrast, Carey 24 and UTSCC 2 cells rarely adhered to HUVECs (2 and 0 events, respectively). Binding of UTSCC 24A and 24B cells to HUVECs with and without pre-incubation with the E-selectin antibody was not significantly influenced by pretreatment with pronase (Figure 3A). In fluorescence-activated cell sorting (FACS) analysis, only 10% of UTSCC 24A and 5% of UTSCC 24B cells bound to the rhE-sel fusion protein and UTSCC 2 and Carey 24 cells did not bind at all. However, 80% to 100% of UTSCC 2, UTSCC 24A, UTSCC 24B, and Carey 24 cells bound to the rhP-sel fusion protein in FACS (Figure 2B). SLeX (CD15s) was expressed by 22% of UTSCC 24A and 29% of UTSCC 24B cells, but not by Carey 24 and UTSCC 2 cells (Figure 2B). Canonical selectin ligand sLeA (CA19-9) was not detected in the HNSCC cells. Static rhE-sel fusion protein binding and sLeX expression were slightly modified by pronase treatment in UTSCC 24A, but not in UTSCC 24B cells (Figure 3B).

### 2.3. Tumor Growth and Metastatic Potential of HNSCC Grown in SCID Mice

**Figure 4:**
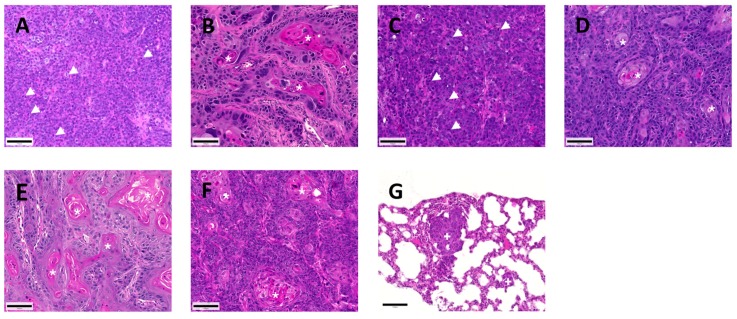
Hematoxylin-eosin (HE)-stained primary tumors and lung metastases of HNSCC cells grown in severe combined immunodeficient (SCID) mice. Note the poorly differentiated UTSCC 2 (**A**) and 24A (**C**) tumors with numerous mitoses (🠜) and immature cells, whereas UTSCC 16A (**B**), 24B (**D**), 60A (**E**), and Carey 24 (**F**) primary tumors were more differentiated with keratin pearl formation (🞳). Only UTSCC 24A cells produced histologically detectable lung metastases (**G**,✚). Scale bar: 100 µm.

**Figure 5:**
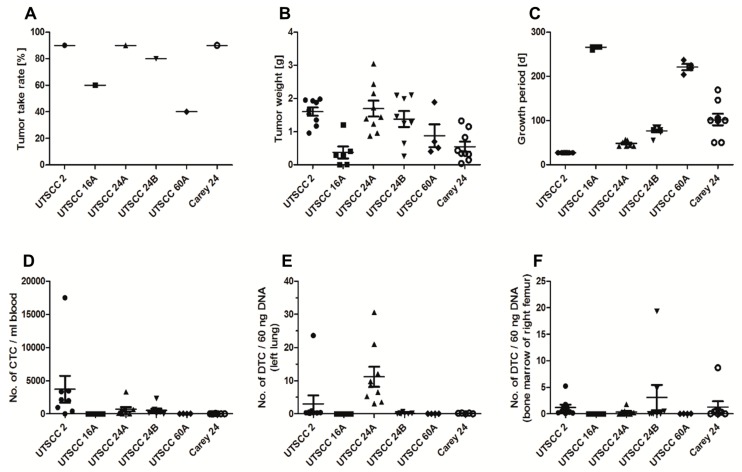
Vertical scatter plots with mean of primary tumor growth and metastasis of HNSCC cells in SCID mice (• UTSCC 2, ■ UTSCC 16A, ▲UTSCC 24A, ▼ UTSCC 24B, ◆ UTSCC 60A, o Carey 24). Tumor take rates (**A**) varied between 40% and 90%, with acceptable take rates of UTSCC 2, UTSCC 24A, UTSCC 24B, and Carey 24 cells. Tumor weights (**B**) of UTSCC 16A, UTSCC 60A, and Carey 24 primary tumors were very low. Growth periods (**C**) of UTSCC 16A and UTSCC 60A tumors were extremely long. Circulating tumor cells (CTCs) in the blood (**D**), and disseminated tumor cells (DTCs) in left lungs (**E**) and in bone marrow of the right femur (**F**) were quantified by Alu-PCR. UTSCC 2, UTSCC 24A, and UTSCC 24B showed considerable numbers of CTCs in blood (**D**). Mice injected with UTSCC 24A cells exhibited significant numbers of DTCs in the lung (**E**).

All tested HNSCC cells were engrafted in SCID mice when co-injected with Matrigel, but tumor histology, take rates, growth behavior, and metastasis formation varied considerably (Figure 4 and Figure 5). Take rates described the percentage of mice that developed primary tumors (Figure 5A). Days from injection to sacrification were defined as the growth period (Figure 5B). Metastasis formation was assessed by the number of tumor cells in the blood, lung, and bone marrow and by histological analysis of the left lungs (Figure 5D–F).

UTSCC 2 and 24A cells produced undifferentiated tumors with many mitoses and immature cells (Figure 4A,C). The other HNSCC cells produced more differentiated primary tumors with keratin pearl formation (Figure 4B,D–F). Only UTSCC 24A cells produced histologically detectable lung metastases (Figure 4G). Histology, growth behavior, and metastasis formation were not associated with originating sites of the cell lines divided in the oral cavity, oropharynx, and larynx according to TMA samples (Table 1).

UTSCC 2 cells produced tumors in nine out of ten mice in four weeks (Figure 5A,C). The mean tumor weight was 1.6 g (Figure 5B). Mice inoculated with UTSCC 2 cells exhibited the highest number of circulating tumor cells (CTCs) in blood (3962 ± 2657 CTCs/mL blood) and the second highest number of disseminated tumor cells (DTCs) in the lung (3.0 ± 2.6 DTCs/60 ng DNA). However, this high mean value was predominantly caused by one outlier and in the histological analysis, lung metastases were not detected. The mean of DTCs in bone marrow was 1.2 ± 1.6 DTCs/60 ng DNA (Figure 5D–F).

UTSCC 24A cells developed tumors in nine out of ten mice, with a mean tumor weight of 1.69 g after seven weeks (Figure 5A–C). The number of CTCs in blood was the second highest, with a value of 690 ± 342 CTCs/mL blood (Figure 5D). DTCs in the lung were the highest in UTSCC 24A (11.2 ± 3.0 DTCs/60 ng DNA) (Figure 5E). Upon histological examination, only UTSCC 24A showed histologically detectable metastases (1793 ± 723 lung metastases, Figure 4C). The number of DTCs in bone marrow was less than 1 DTC/60 ng DNA (Figure 5F).

UTSCC 24B cells produced tumors in eight out of ten mice (Figure 5A). The number of CTCs in blood was high (541 ± 265 CTCs/mL blood), but the number of DTCs in the lung was less than 1 DTC/60 ng DNA (Figure 5D,E). DTCs in bone marrow were detected in 55% of UTSCC 24B mice, showing the highest mean value of 3.1 ± 2.4 DTCs/60 ng DNA (Figure 5F).

Nine out of ten mice inoculated with Carey 24 cells developed primary tumors with a growth period of 102 days and mean tumor weight of 0.54 g (Figure 5A–C). Numbers of CTCs in blood and DTCs in the lung were biologically insignificant (Figure 5D,E). The mean of DTCs in bone marrow was 1.3 ± 2.9 DTCs/60 ng DNA (Figure 5F).

The take rate of UTSCC 16A was only four out of ten mice with small tumors (mean weight: 0.37 g) and a long growth period (mean: 266 days) (Figure 5A–C). Numbers of CTCs in blood and DTCs in the lung and bone marrow were less than 1 CTC/mL blood and 1 DTC/60 ng DNA, respectively (Figure 5D–F).

Xenograft tumors were developed in four out of ten UTSCC 60A-bearing mice with low tumor weights (mean: 0.55 g) and a long growth period (mean: 221 days) (Figure 5A–C). Numbers of CTCs in blood and DTCs in the lung and bone marrow were less than 1 CTC/mL blood and 1 DTC/60 ng DNA, respectively (Figure 5D–F).

### 2.4. Immunohistochemistry

The results of immunohistochemical analyses are summarized in Table 2.

Whilst UTSCC 2 and UTSCC 24A primary tumors reacted moderately with anti E-cadherin, all other HNSCC xenograft tumors showed strong E-cadherin expression. In contrast, N-cadherin expression was weak in primary tumors, except for UTSCC 2 and 24A primary tumors, which showed a moderate reaction with the anti N-cadherin antibody.

SLeA (CA19-9) was only detected in tumors of three cell lines, namely UTSCC 24A, UTSCC 24B, and UTSCC 60A, and the other HNSCC tumors did not express sLeA.

SLeX (CD15s) expression was predominantly found in UTSCC 24A xenograft tumors. The other primary tumors exhibited, at most, 5% sLeX-positive cells. UTSCC 2 tumors did not express sLeX.

Primary tumors of all HNSCC cells reacted strongly with anti CD44. UTSCC 2 and 24A tumors showed strong staining of circumscribed areas (about 25% of tumor tissue). Tumors of the other HNSCC cells were CD44 positive, except for keratin pearls.

CD24 expression was detected in keratin pearls of UTSCC 24B, 16A, and 60B primary tumors. Primary tumors of UTSCC 24A weakly expressed CD24, UTSCC 24A lung metastases, and UTSCC 2, and Carey 24 primary tumors did not express CD24.

UTSCC 16A, 60A, and Carey 24 xenograft tumors showed moderate staining, and UTSCC 24B weak staining, with anti ICAM. Single ICAM-positive cells could be found in UTSCC 24A tumors. UTSCC 2 xenograft tumors did not express ICAM.

L1CAM was strongly immunoreactive in UTSCC 16A, 24B, and 60A tumors. UTSCC 2 was L1CAM negative, and UTSCC 24A and Carey 24 showed weak L1CAM expression.

UTSCC 2 and UTSCC 24A tumors did not express ITGA5, and xenograft tumors of the other HNSCC cells showed weak ITGA5 expression.

All HNSCC xenograft tumors of the six cell lines reacted with anti ITGB1 and ITGB4 without considerable differences.

CAM expression of xenograft tumors did not correlate with the originating site of cell lines divided in the oral cavity, oropharynx, and larynx, according to TMA samples (Table 1).

### 2.5. Tissue Microarray

**Figure 6:**
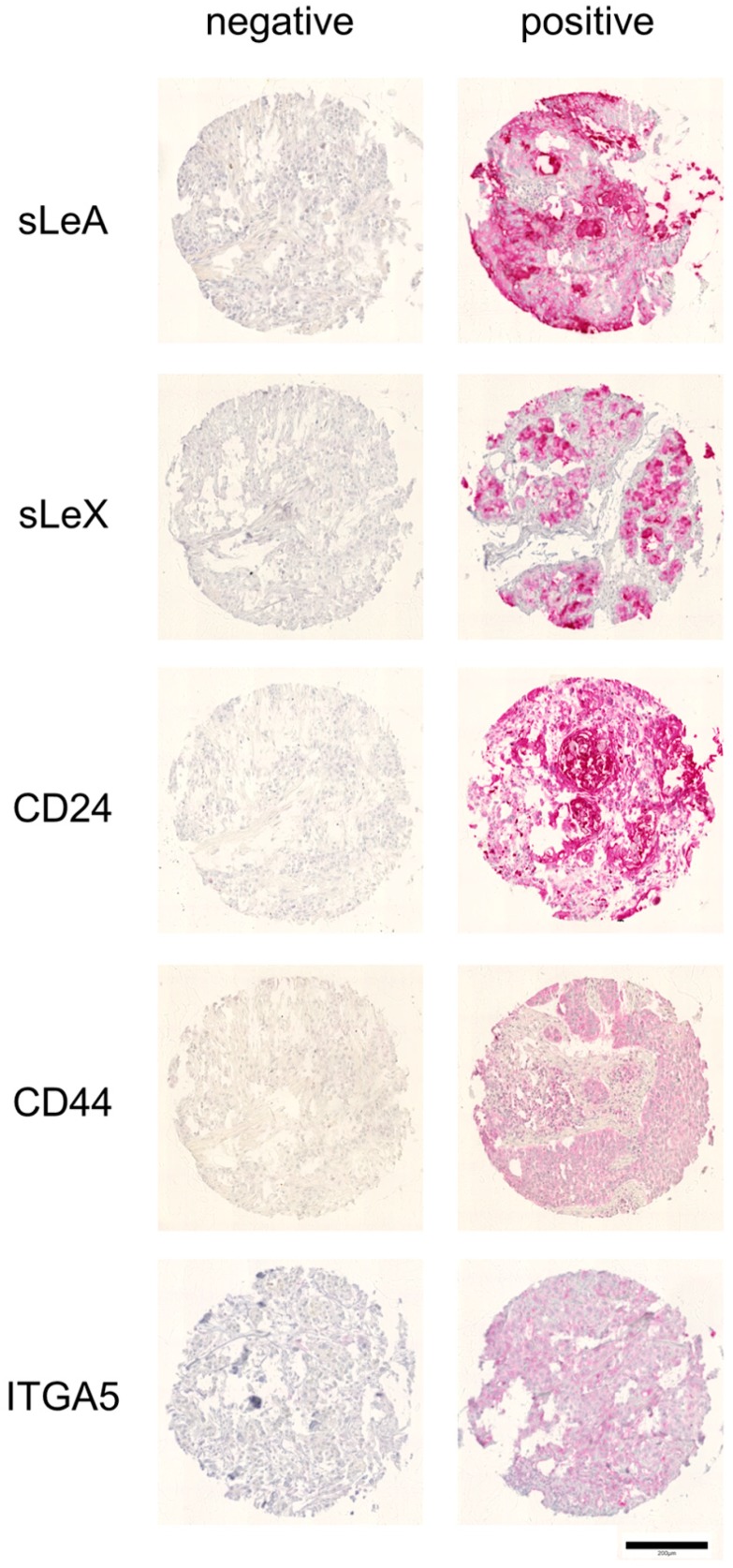
Representative negative and positive tumor samples of immunohistochemical stains of the tissue microarrays (TMA). Scale bar: 200 µm.

The TMA comprised a total of 453 cases of head and neck squamous cell carcinomas, but the evaluation, revealed less cases that were interpretable for immunohistochemical analyses. The decrease in sample size resulted from the absence of tissue on the respective TMA section or a lack of unequivocal tumor cells in the arrayed sample. Figure 6 shows examples of positive and negative tumor samples stained with anti sLeA, sLeX, CD24, CD44 and ITGA5.

The expression of sLeA was analyzed in a total of 198 HNSCCs, including 62 cancers of the oral cavity, 62 of the oropharynx, and 74 of the larynx. SLeA expression did not show significant differences between TNM (tumor, node, metastases) and UICC (Union International Contre le Cancer) stage, respectively, in all analyzed subgroups, with 17% to 21% sLeA-positive tumors in each group.

Fifty-one of 200 HNSCC samples (25%) expressed sLeX. Though immunostaining exhibited significant differences between the four UICC stages in a total of 200 HNSCC cases, sLeX positivity did not linearly correlate with an advanced tumor stage. In the subgroups, 25% of cancers of the oropharynx, 16% of cancers of the oral cavity, and 30% of larynx carcinomas were sLeX positive. The expression of sLeX was significantly related to higher pT)-stages in 65 cancers of the oropharynx (pT1+2 vs. pT3+4; *p* = 0.04), whereas analyses of 56 cancers of the oral cavity and 79 larynx carcinomas did not reveal any statistically significant results.

CD24 expression was studied in 272 HNSCC cancers, including 85 cancers of the oral cavity, 82 cancers of the oropharynx, and 105 cancers of the larynx. In total, 82% of all evaluated HNSCC samples, 91% of oropharyngeal cancers, 82% of cancers of the oral cavity, and 74% of laryngeal cancers were positive for CD24. CD24 showed a significant association with pT- and UICC-stage (*p* = 0.0039 and 0.0057, respectively) for cancers of the oral cavity. Association of the subset of oral squamous cell carcinoma (OSCC) with recurrence-free and overall survival was not noted. It was unrelated to clinical and histological parameters and recurrence-free or overall survival of the whole HNSCC cohort, as well as the subsets of oropharynx and larynx.

CD44 expression was analyzed in a total of 241 HNSCC cancers, including 86 cancers of the oral cavity, 71 of the oropharynx, and 84 of the larynx. In total, 67% of all HNSCC cancers, 67% of cancers of the oral cavity, 70% of cancers of the oropharynx, and 63% of cancers of the larynx expressed CD44. CD44 expression was not associated with the pT-, pN-, or pM-stage; UICC stage; and recurrence-free or overall survival in overall or subset analyses.

ITGA5 expression was analyzed in a total of 195 HNSCC cancers, including 59 cancers of the oral cavity, 68 of the oropharynx, and 68 of the larynx. The percentage of ITGA5-positive samples was 10% in cancers of the oral cavity, 16% in cancers of the oropharynx, and 19% in cancers of the larynx. ITGA5 expression was not associated with pT- or pN-stage, UICC stage, and recurrence-free or overall survival in overall or subset analyses. Because of the small sample size, association between ITGA5 and pM-stage (*p* = 0.0051) for cancers of the oral cavity was biologically insignificant.

## 3. Discussion

Distant hematogenous metastases represent one important cause of cancer-related death in patients with HNSCC [1]. Because of this, it is of great interest to gain deeper insight into mechanisms of metastatic spread. Therefore, models of the spontaneous metastasis formation of human head and neck cancer have to be developed.

In our xenograft model, UTSCC 2 and 24A cells producing undifferentiated xenograft tumors showed the highest take rates, shortest growth periods, highest tumor weights, and numerous mitoses in the tumors reflecting their high proliferative activity. However, in the following experiments, the primary tumor take rate of UTSCC 24A cells was significantly lower. UTSCC2 and 24A tumors also displayed the highest N-cadherin and lowest E-cadherin expression. This cadherin pattern has been shown to correlate with tumor progression and metastasis in squamous cell carcinoma (SCC) [15,16,17,18,19]. Furthermore, low E-cadherin expression in the primary tumor is suspected to predict the metastatic potential of patients with HNSCC [20].

The highest numbers of CTCs and DTCs, respectively, were found in mice inoculated with UTSCC 2, 24A, and 24B cells, which also showed high take rates and short growth periods, indicating that high numbers of CTCs/DTCs go along with aggressive tumor growth in xenograft models. Considering previous studies, numbers of CTCs or DTCs in bone marrow seem to have a clinical prognostic relevance in patients with SCC [21,22,23,24].

UTSCC 2 displayed the highest number of CTCs in blood, but showed less DTCs in the lung than UTSCC 24A cells and did not produce microscopically visible lung metastases. This result indicates that UTSCC 2 cells disperse from primary tumors and intravasate, but are less qualified to adhere and extravasate at distant sites. In contrast, UTSCC 24A cells produced many DTCs in the lung and histologically verified lung metastases, suggesting that these cells can adhere to the endothelium and transmigrate over the endothelial barrier. At this part of the metastatic cascade, heterotypic interactions between circulating tumor cells (CTCs) and endothelial cells represent the basic step that leads to tumor cell extravasation and are mediated by a variety of cell adhesion molecules (CAMs) based on the model of the leukocyte adhesion cascade [2,9,10,25,26]. In particular, the selectin family mediates the initial attachment of leukocytes to endothelial cells [27]. Selectin binding properties of the HNSCC cells were examined under static (FACS) and dynamic (flow assay) conditions because it is well-known that shear stress influences selectin-receptor-ligand bonds [28,29]. All tested cells exhibited static P-selectin binding, but they did not adhere to the rhP-sel fusion protein at all in flow assays. Considerable static E-selectin binding was only detected in UTSCC 24A and 24B cells, which also stuck to the rhE-sel fusion protein under flow. In earlier xenograft studies, selectin deficiency in mice caused a reduction of lung metastases, but increased the number of CTCs [30]. The lack of E-selectin binding sites of UTSCC 2 cells may explain the high number of CTCs next to the absence of histologically detected lung metastases in the mouse model.

The classical (canonical) binding partners of selectins are carbohydrate structures whose minimal recognition motifs are represented by sialyl Lewis X (sLeX) and sialyl Lewis A (sLeA) [28]. Whereas sLeA was not detected on HNSCC cells, sLeX was found on UTSCC 24A and 24B cells which also bound to the rhE-sel fusion protein in FACS. Concerning the nature of the selectin binding sites, the results of pronase pretreatment indicate that selectin binding glycans are linked to proteins and lipids in UTSCC 24A cells, but only to lipids in UTSCC 24B cells.

In agreement with the in vitro results, UTSCC 24A xenograft tumors also expressed sLeX most strongly. However, sLeX-positive cells were found in all xenograft tumors except for UTSCC 2 and sLeX expression was independent of originating sites of the cell lines. TMA analysis showed a correlation between sLeX expression and tumor stage in the subgroup of oropharyngeal cancer, but not in the other subgroups or in the overall analysis. Studies evaluating sLeX in HNSCC are limited and the results are inconsistent. Therefore, sLeX is described as a negative prognostic marker in one study, as prognostically insignificant in another, and as a potential metastatic marker in a further study [31,32,33,34].

Previous studies have identified sLeX-modified CD24 as a functional P-selectin ligand that can promote rolling and tumor cell colonization to the lung in lung adenocarcinoma cells [35,36]. CD24 is a cell surface protein that acts as a ligand for P-selectin. It was shown to be associated with cisplatin resistance and was thus correlated with a poor clinical outcome in laryngeal carcinoma [37]. In our TMA analysis, CD24 was only associated with tumor stage in the subgroup of cancers of the oral cavity. A correlation with overall survival or metastasis was not proved. In the xenograft model, CD24 was observed on UTSCC 16A, 24A, 24B, and 60A tumors and was not associated with the metastatic capability or originating site. However, UTSCC 24A cells deriving from the tongue co-expressed CD24 and sLeX in vitro. As only these cells produced visible lung metastases, sLeX-modified CD24 could be involved in the metastatic properties of these cells.

Though the other canonical selectin ligand sLeA is described as a potential metastatic marker for HNSCC in one previous study, immunohistochemical analysis of xenograft tumors and TMAs did not show a correlation between sLeA and clinical and histological parameters [34].

A specialized sialofucosylated glycoform of CD44 (HCELL, Hematopoietic Cell E/Lselectin Ligand) is also a very potent E-selectin ligand [38]. CD44 is involved in cell-cell and cell-matrix adhesion and acts as cancer stem cell and prognostic marker in several tumor entities [35,39,40]. Numerous immunohistochemical studies have sought a relationship between CD44 expression and various clinical parameters in HNSCC, but the reported data are largely conflicting [41]. In this study, a relevant association between CD44 expression and tumor progression or metastasis was not found in the mouse xenograft model and in patient material (TMA).

In addition to selectin-dependent adhesion, selectin-independent adhesion and extravasation has been observed [42,43]. These observations are corroborated by the fact that the knockout of selectins often reduces, but does not totally prevent, metastasis formation [30,44,45]. The family of integrins has also been associated with cancer progression [46]. Integrins mediate cell adhesion, extravasation, and cell signaling of tumor cells and malignant tumors frequently present altered integrin expression [47]. In our study, ITGA5 was expressed at low to undetectable levels on metastatic UTSCC 24A cells and corresponding xenograft tumors. ITGA5 expression has been shown to predict the clinical outcome in HNSCC in previous studies, but was not verified as a prognostic marker in our TMA analysis [48,49,50].

## 4. Materials and Methods

### 4.1. Cell Lines

Human head and neck squamous cell carcinoma cell lines (HNSCC), namely UTSCC 2, UTSCC 16A, UTSCC 24A, UTSCC 24B, UTSCC 60A, and Carey 24, were a generous gift from Reidar Grenmann (University of Turku, Finnland) and Thomas Carey (University of Ann Arbor Michigan, USA), respectively. The cell lines are described in Table 1.

Cells were cultured in vitro under standard cell culture conditions (37 °C, 100% relative humidity, 5% CO_2_) in RPMI medium (Gibco/Life Technologies, Karlsruhe, Germany) supplemented with 10% heat-inactivated fetal bovine serum (FBS, Gibco), 2 mM L-glutamine (Gibco), 100 U/mL penicillin, and 100 µg/mL streptomycin (Gibco).

### 4.2. Flow Cytometry

E- and P-selectin (724-ES, 137-PS, rhE- and rhP-selectin/IgG-Fc chimera, R&D Systems, Minneapolis, Minnesota, USA) binding sites were analyzed with and without pronase pretreatment. Pronase from *Streptomyces griseus* (Roche Diagnostics, Mannheim, Germany) hydrolyzes peptide bonds within proteins. Unaltered selectin binding after pronase pretreatment indicates that binding is mediated by glycolipids rather than glycoproteins.

In this study, 1 µL rhE-selectin- or P-selectin-Fc-chimera, or Fc-control (1 mg/mL), respectively, were prepared with 100 µL FACS-buffer + 1 mM Ca^2+^ + 1 mM Mg^2+^, and complexed by incubation with 1.25 µL goat anti-human-IgG-PE (0.5 mg/mL). For pronase pretreatment, a total of 1 × 10^6^ cells in serum-free RPMI medium were incubated with 100 µg pronase for 45 min at 37 °C.

The antibodies employed for FACS are specified in Table 3. Tumor cells were marked dead or alive by propidium iodide (Sigma–Aldrich, Hamburg, Germany) staining and analyzed using a Cube 8 (Partec, Münster, Germany) cytometer and FCS Express 4 software (De Novo Software, Los Angeles, CA, USA).

### 4.3. Laminar Flow Adhesion Assay

The adhesion of UTSCC 2, UTSCC 24A, UTSCC 24B, and Carey 24 cells on rhE-selectin and P-selectin (R&D Systems) and stimulated (10 ng/mL IL-1α, PeproTech, Hamburg, Germany) HUVECs was analyzed under physiological flow conditions in ibidiTreat µ-slide IV0.4 flow chambers, as previously described [51]. The applied shear rate was 0.5 dyn/cm^2^. Stimulated HUVECs were incubated with an adhesion blocking E-selectin mAb (BioLegend, San Diego, CA, USA) prior to the experiment [51]. Furthermore, cancer cells were pretreated with pronase, as described in the section above.

### 4.4. SCID Mouse Experiments

Animal experiments were conducted according to the UKCCR (United Kingdom Coordinating Committee on Cancer Research) guidelines for the welfare of animals in experimental neoplasia [42] and were approved by the local animal care committee and assigned the project No. G09/70. In this study, 1 × 10^6^ HNSCC cells were suspended in 100 µL cell culture medium and blended 1:1 with Matrigel (BD Bioscience, Bedford, MA, USA). Cells were injected subcutaneously between the scapulae of pathogen-free BALB/c severe combined immunodeficient (SCID) mice. Each group of mice included ten animals.

The mice bearing HNSCC were sacrificed when the tumor reached maximal growth (up to 2 cm^3^) or started to ulcerate. Primary tumor, blood, lung, and bone marrow samples of the right femur were extracted for further examinations.

### 4.5. Quantification of Circulating (CTC) and Disseminated Tumor Cells (DTC) by Alu-PCR

Amounts of CTCs in blood and DTCs in the right lung and bone marrow were determined by a real-time polymerase chain reaction [42,52,53]. DNA concentrations of all samples were quantified using a NanoDrop spectrophotometer (Peqlab, Erlangen, Germany). QPCR was performed with established human-specific *Alu*-primers [42,52,53]. A total of 2 μL total DNA (60 ng lung/bone marrow-DNA; 20 ng blood-DNA) was used for each qPCR. Numerical data were determined against a standard curve, as previously described [54].

### 4.6. Histology

Primary tumors and left lungs were processed to paraffin wax and stained with hematoxylin-eosin (HE), and the number of pulmonary metastases was determined as previously described [55].

### 4.7. Immunohistochemistry

Antibodies used for immunohistological analyses of xenograft tumors and TMAs and respective pretreatment are described in Table 4. Corresponding isotype controls served as negative controls. After deparaffinization and demasking, non-specific binding was blocked by 10% normal rabbit serum (DAKO). Immunohistochemical detection of E-cadherin, N-cadherin, and ICAM was performed using the K5005 detection kit (DAKO). Other antibodies were detected by the streptavidin-alkaline phosphate kit (ABC-AP; Vector Laboratories). Immunohistochemistry was assessed by the staining intensity and fraction of positive tumor cells (Table 2).

### 4.8. Tissue Microarrays

HNSCC material was collected from surgically removed tissue by adhering to guidelines of the local ethical review board and after written informed consent from the patients. The usage of archived diagnostic left-over tissues for manufacturing of tissue microarrays and their analysis for research purposes as well as patient data analysis has been approved by local laws (HmbKHG, §12,1) and by the local ethics committee (Ethics commission Hamburg, WF-049/09). All work has been carried out in compliance with the Helsinki Declaration. It comprised 453 cases of head and neck squamous cell carcinomas, including 222 oral (49%), 126 pharyngeal (27.8%), and 105 laryngeal (23.2%) tumors. Detailed clinical and pathological data have been described previously [56]. Tissue samples of HNSCC were fixed in buffered 4% formalin and processed as previously described [57,58]. Staining was evaluated by the staining intensity and fraction of positive tumor cells for each tissue spot. These two parameters provided an overall score dividing negative, weak, moderate, and strong scores, as described previously [59]. According to these results, the samples were grouped into tumors with negative staining (negative score) and positive staining (weak, moderate, and strong score) and based on their localization for the statistical analysis of tissue microarrays.

### 4.9. Statistical Analyses

Data analyses of in vitro experiments and xenograft studies were carried out using GraphPad Prism^TM^ software (GraphPad^TM^, San Diego, CA, USA). The results of laminar flow assays were compared with an analysis of variance (one-way ANOVA) and Bonferroni’s posttest.

The statistical analysis of TMAs was conducted using JMP 10.0 software (SAS institute Inc., Cary, NC, USA). All p-values were two-sided and *p*-values < 0.05 were considered significant. To study the relationship between CAM expression and clinical–pathological parameters, a contingency table analysis and chi-squared test (Likelihood) were used. Survival curves were calculated via the Kaplan–Meier method and compared using the Log-rank test. Cox regression was used to assess the independence of CAM expression, including the parameters pT-stage, pN-stage, pM-stage, and clinical stage (UICC 7th edition; 2009).

## 5. Conclusions

Analyses of CAMs and their ligands of the leukocyte adhesion cascade in xenograft tumors and patient material (TMA) did not reveal a distinct prognostic or metastatic marker for HNSCC. In TMA, CD24 only showed a correlation to tumor stage in the subgroup of oral cavity and the selectin ligand sLeX in the subgroup of oropharyngeal cancer, respectively. Furthermore, sLeX–E-selectin interactions played a role in the in vitro adhesion of UTSCC 24A and 24B cells to endothelial cells. Further cell adhesion molecules which are clearly involved in HNSCC metastasis have to be identified in order to provide novel anti-metastatic therapeutic approaches.

## Figures and Tables

**Table 1:** HNSCC cell lines used for in vitro and xenograft analyses.

Cell Line	Sex	Age	Primary Tumor Location	TNM	Specimen Origin	Grade
UTSCC 2	M	60	Oral cavity (base of mouth)	T_4_N_1_M_0_	pri	
UTSCC 16A	F	77	Oral cavity (tongue)	T_3_N_0_M_0_	pri	G3
UTSCC 24A	M	41	Oral cavity (tongue)	T_2_N_0_M_0_	pri	G2
UTSCC 24B	M	41	Derived from metastatic site: neck; pri: oral cavity (tongue)		met	G2
UTSCC 60A	M	59	Oropharynx (left tonsil)	T_4_N_1_M_0_	pri	G1
Carey 24	M	57	Larynx (true vocal cord)	T_1_N_0_M_0_	rec	

**Table 2:** Immunohistochemical analyses of HNSCC xenograft tumors.

Antibodies	UTSCC 2	UTSCC 16A	UTSCC 24A		UTSCC 24B	UTSCC 60A	Carey 24
	Primary Tumor	Primary Tumor	Primary Tumor	Lung Metastases	Primary Tumor	Primary Tumor	Primary Tumor
**E-cad**	++	+++ a	++/+++	++	+++ a	+++ a	+++ a
**N-cad**	+++	+	++	+	+	+	+
**sLeA (CA19-9** **)**	-	-	+++ (5–25)	+++ b	+++ (<10)	+/++	-
**sLeX (CD15s)**	-	+++ b	+ (80) +++ (5)	-	+++ (5–10)	+++ b	+++ b
**CD 44**	+++ (25)	+++ a	+++ (25)	+++ b	+++ a	+++ a	+++ a
**CD 24**	-	++ c	+	-	++ c	++ c	-
**ITGA5**	-	+ a	-	-	+ a	+/++ a	+ a
**ITGb1**	+	+	+	+	+	++	+
**ITGb4**	+	+	+	+	+	+	+
**ICAM**	-	++ a	+++ b	+++ b	++ b	++/+++ a	++ a
**L1CAM**	-	++	+	+	++ (30–50)	+++ (50)	+

a—Keratin pearls were negative; b—Positivity in single cells; c—Positivity in keratin pearls. Staining intensity: - negative, + weak, ++ moderate, +++ strong; in brackets: percentage of positive tumor cells.

**Table 3:** Commercial antibodies used for FACS.

ALCAM (105902, R&D systems, Minneapolis, USA)
CA19-9 (sLeA) (121SLE, Novus Biological, Littleton, CO, USA)
CD11a (HI111, BioLegend, London UK)
CD11b (ICRF44, eBioscience, Waltham, MA, USA)
CD15s (sLeX) (FH6, BioLegend)
CD44 (B-F24, Diaclone, Besancon Cedex, France)
CD46 (TRA-2-10, BioLegend)
CD24 (eBioSN3, eBioscience)
CD162 (FLEG, eBioscience)
EpCAM (1B7, eBioscience)
ICAM-1 (HA58, eBioscience)
ITGAV (NKI-M9, BioLegend)
ITGA4 (9F10, BioLegend)
ITGA5 (P1D6, eBioscience)
ITGA6 (GoH3, BioLegend)
ITGB1 (TS2/16, eBioscinece)
ITGB2 (TS1/18, BD Bioscience, Heidelberg, Germany)
ITGB3 (VI-PL2, BioLegend)
ITGB4 (439-9B, eBioscience)
ITGB5 (KN52, eBioscience)
ITGB7 (473207, R&D systems)
L1CAM (eBio5G3, eBioscience)

**Table 4:** Commercial antibodies and pretreatment used for immunohistochemical analyses.

Antibodies	Pretreatment
CA19-9 (sLeA) (121SLE, abcam, Cambridge, UK)	0.1% trypsin in TBS, 5 min
CD15s (sLeX) (CSLEX1, BD Pharmingen, Heidelberg, Germany)	Citrate buffer, steamer, 10 min, 121 °C
CD24 (SWA11, kindly provided by Prof. Peter Altevogt, German Cancer Research Centre, Heidelberg, Germany)	Fast enzyme (Zytomed, Berlin, Germany) in TBS, 10 min
CD44 (G44-26, BD Pharmingen)	S1699 (DAKO), steamer, 10 min, 121 °C
E-cadherin (NCH 38, DAKO, Glostrup, Denmark)	S1699 (DAKO), water bath, overnight, 85 °C
ICAM-1 (G-5, Santa Cruz, CA, USA)	S1699 (DAKO), microwave
ITGA5 (EPR7854, abcam)	S1699 (DAKO), steamer, 10 min, 121 °C
ITGB1 (4B7R, abcam)	Fast enzyme (Zytomed) in TBS, 10 min
ITGB4 (439-9B, abcam)	S1699 (DAKO), microwave
L1CAM (UJ127, abcam)	EDTA, microwave
N-cadherin (6-G11, DAKO)	0.1% trypsin in TBS, 5 min
